# Making Sense of Shoulder Exercise: Measuring the Accuracy of an Artificial Intelligence Model to Classify Shoulder Exercise via Wearable Sensors Among People With and Without Rotator Cuff Tendinopathy

**DOI:** 10.1002/ejsc.70167

**Published:** 2026-04-08

**Authors:** Josh Naunton, Yanran Jiang, Rodrigo Bini, Dawson Kidgell, Kim Bennell, Terry Haines, Dana Kulić, Peter Malliaras

**Affiliations:** ^1^ Department of Physiotherapy, School of Primary and Allied Health Care, Faculty of Medicine, Nursing and Health Science Monash Musculoskeletal Research Unit (MMRU) Monash University Melbourne Australia; ^2^ Department of Rural Allied Health La Trobe University Rural Health School Bendigo Australia; ^3^ Physiotherapy & Exercise Physiology, Allied Health and Continuing Care Bendigo Health Bendigo Australia; ^4^ Department of Mechanical and Aerospace Engineering, Faculty of Engineering Monash University Melbourne Australia; ^5^ Department of Physiotherapy, School of Primary and Allied Health Care, Faculty of Medicine, Nursing and Health Science Monash Exercise Neuroplasticity Unit Monash University Melbourne Australia; ^6^ Centre for Health, Exercise and Sports Medicine, Department of Physiotherapy, Faculty of Medicine Dentistry and Health Sciences, Level 7 The University of Melbourne Melbourne Australia; ^7^ Rehabilitation, Ageing and Independent Living Research Centre, School of Primary and Allied Health Care, Faculty of Medicine, Nursing and Health Science Monash University Melbourne Australia

**Keywords:** exercise, IMU, machine learning artificial intelligence, rotator cuff tendinopathy, wearables

## Abstract

This study aimed to compare the accuracy of machine learning classification for three commonly prescribed shoulder exercises in people with and without rotator cuff tendinopathy. Eighteen participants with rotator cuff tendinopathy (mean age 54.2, SD 13.2; 50% female), followed by eighteen matched controls completed a laboratory‐based shoulder strength testing protocol. Three exercises were performed (shoulder press, lateral raise and bent over row) while wearing three inertial measurement (IMU) sensors (Axivity, Ax6 ‐ 3 axis accelerometry and gyroscope at 100 Hz and 1000°/sec respectively) positioned on the wrist arm and trunk. Data were analysed and accuracy was compared between common machine learning algorithms for those with rotator cuff tendinopathy and healthy matched controls in a subject‐dependent and subject‐independent model. The best accuracy scores for the subject‐dependent results were achieved by a random forest algorithm; 96.12% (3‐sensor combined system) for those with rotator cuff tendinopathy. For the subject‐independent results best accuracy scores were achieved by a convolutional neural network algorithm; 94.55% (3‐sensors) for the healthy controls without shoulder pain. K‐fold cross validation confusion matrix results by exercise type for the entire cohort show 97% accuracy (shoulder press), 95.5% (lateral raise) and 90.7% (bent over row) (3‐sensors, CNN subject‐independent analysis). Machine learning classification of 3 different shoulder exercises in people with rotator cuff tendinopathy and matched healthy controls demonstrate most accurate results using a CNN algorithm for subject‐independent analysis and a RF algorithm for subject‐dependent analysis. Results were similar for both those with rotator cuff tendinopathy and their matched healthy controls.

## Highlights

- Shoulder exercise can be classified with good accuracy scores using an inertial measurement unit (IMU) accelerometer and gyroscope sensor placed on the wrist.
- Subject‐dependent and subject‐independent models demonstrate comparable results depending on the chosen machine learning algorithm.
- Exercise classification based on a wearable IMU shows similar results between those with rotator cuff tendinopathy and matched healthy controls without shoulder pain.

## 1 Background

Shoulder pain is a prevalent musculoskeletal condition affecting up to 27% of adults aged under 70 at any one point in time (Luime et al. 2004). The most common cause of shoulder pain is rotator cuff tendinopathy, accounting for up to 70% of all musculoskeletal shoulder pain (van der Windt et al. 1995). Clinical practice guidelines recommend progressive exercise over 6–12 weeks as the first line treatment for rotator cuff tendinopathy (British Orthopaedic Association 2014; Diercks et al. 2014; Hopman et al. 2013; Klintberg et al. 2015; Littlewood et al. 2019). Despite the recommendations for exercise treatment, recent trials have reported low exercise adherence rates, potentially impacting on the effectiveness of exercise treatment (Clausen et al. 2021; Hopewell et al. 2021; Liaghat et al. 2020; Littlewood et al. 2015). Recording and monitoring exercise adherence currently relies on direct supervision or video recording as a gold standard measure with most trials relying on self‐reported exercise diaries. Few trials use technology to measure what exercise has been performed (Clausen et al. 2021; Hopewell et al. 2021; Liaghat et al. 2020; Littlewood et al. 2015; D. Burns et al. 2021). Measuring exercise adherence via objective means will improve our understanding of the most effective or optimal exercise prescription variables for rotator cuff tendinopathy (Littlewood et al. 2015; Malliaras, Johnston, et al. 2020; Naunton et al. 2020; Page et al. 2016). Exercise prescription is regularly based on one repetition maximum (1RM) strength results, yet there is a lack of normative data for 1RM tests for common shoulder exercises among people with rotator cuff tendinopathy, as isometric hand held dynamometry is commonly utlised (Ingwersen et al. 2017; Larsson et al. 2019; Chezar et al. 2013; Berkovitch et al. 2013; Almeida et al. 2020; Clausen et al. 2017; Akyol et al. 2013).

Recent advances in wearable technology, such as smartwatches, accelerometers, inertial measurement units (IMUs), activity trackers or smartphones may potentially provide a convenient, efficient and acceptable method to objectively monitor exercise adherence. Use of such technology is increasing within healthcare with devices now able to track step count, energy expenditure, maximal oxygen consumption (VO_2_max), sleep, oxygen saturation and even reporting a basic ECG signal (Shei et al. 2022; Li et al. 2022). Various smartwatches (Samsung Galaxy Watch, Garmin, Amazfit, Coros) have recently implemented repetition counting for a limited set of exercises, although this is reliant on manually starting and stopping the exercise recording. This technology often relies on artificial intelligence machine learning algorithms. However, the best machine learning algorithm and its accuracy in classifying shoulder exercise is not known.

Several studies have investigated the use of wearable technology to classify IMU sensor signal by the exercise performed, tracking step count or physical activity (Um et al. 2017; D. M. Burns et al. 2018; Chang et al. 2007; Cheng et al. 2017; Ebert et al. 2017; Morris et al. 2014; Muehlbauer et al. 2011; O'Reilly et al. 2017; Seeger et al. 2011; Shen et al. 2018; Gorzelitz et al. 2020). Despite this, few studies have investigated IMU sensor signal for exercise classification among clinical populations with only two other lab groups doing so among people with rotator cuff tendinopathy (D. Burns et al. 2021; Bavan et al. 2019; Bavan et al. 2020).

This study aims to: (1) compare the concurrent validity and accuracy of various machine learning algorithms' ability to automatically detect and classify recorded IMU sensor signal by exercise (i.e., shoulder press, lateral raise or bent over row) among people with and without rotator cuff tendinopathy; (2) compare the accuracy of IMU sensor placement between the wrist, arm, trunk or a combined 3‐sensor system; and (3) to measure one repetition maximum (1RM) strength of a shoulder press, lateral raise and bent over row for people with rotator cuff tendinopathy compared to matched healthy controls.

## 2 Methods

### 2.1 Experimental Design

This prospective non‐randomised observational matched case‐control study was reported according to the STROBE guidelines (Strengthening the Reporting of Observational Studies in Epidemiology) (Cuschieri 2019). Ethical approval was granted from the University Ethics Committee (No. 27192). Data were gathered from the Biomechanics Laboratory at a partner University campus subsequent to obtaining site approval from their own University Human Research Ethics Committee. All participants gave written informed consent prior to completing the study.

### 2.2 Participants

**FIGURE 1:**
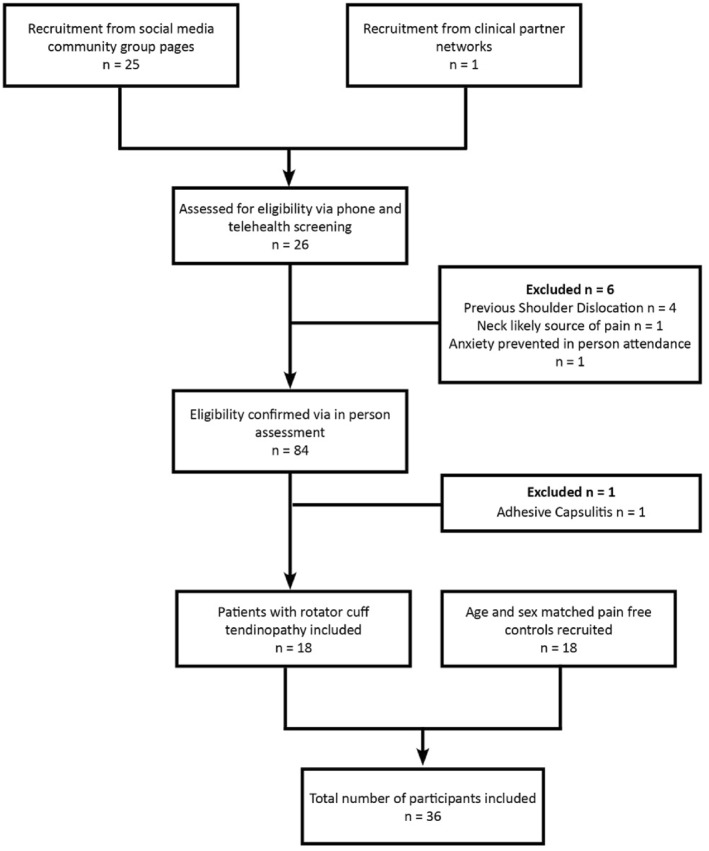
Recruitment flow diagram.

Thirty‐six participants (18 with shoulder pain and 18 matched controls) were recruited from a regional centre (Victoria, Australia) to take part in the trial. We aimed to recruit a minimum of 30 participants (minimum requirement not to be considered inadequate as per the COSMIN guidelines) (Mokkink et al. 2019) to detect a correlation of ≥ 0.6 (*α* = 0.05, power = 0.95), noting that power analysis is not usually applied to machine learning approaches (Rajput et al. 2023; Balki et al. 2019). 18 participants with rotator cuff tendinopathy were recruited via social media and clinical partners (Figure 1). Information regarding the trial was posted in free community and personal social media pages and posters were placed in the waiting rooms of clinical partners. Eligibility and screening were completed via phone call, with video when required in accordance with previously utilised methods (Malliaras, Cridland, et al. 2020). If the diagnosis remained uncertain, participants underwent face to face clinical examination by the primary author (more than 10 years clinical experience) prior to inclusion. Eligibility and exclusion criteria are listed in Table 1. Healthy participants without shoulder pain were recruited via the same social media channels and matched for age (within 2 years) and sex to the shoulder pain group. Participants utilised their dominant hand to match the painful group, for example, if the participant with pain used their dominant hand then the matched participant also used their dominant hand.

**TABLE 1:** Inclusion and exclusion criteria for the study.

Inclusion criteria for participants with rotator cuff tendinopathy	Exclusion criteria (both groups)
Primary presenting complaint of anterolateral shoulder pain (with or without referral into the upper arm) Shoulder pain that is primarily associated with movement, particularly overhead activity or increased shoulder load resistance Preservation of passive shoulder range of movement Age 18 years or older	Cervical spine referred pain Hemiplegic shoulder pain Systemic pathology including inflammatory joint conditions and neoplastic disorders e.g. rheumatoid arthritis Neurological disease affecting the shoulder (e.g. Parkinson's disease) Clinical diagnosis consistent with adhesive capsulitis or glenohumeral osteoarthritis

### 2.3 IMU Sensor Measures

**FIGURE 2:**
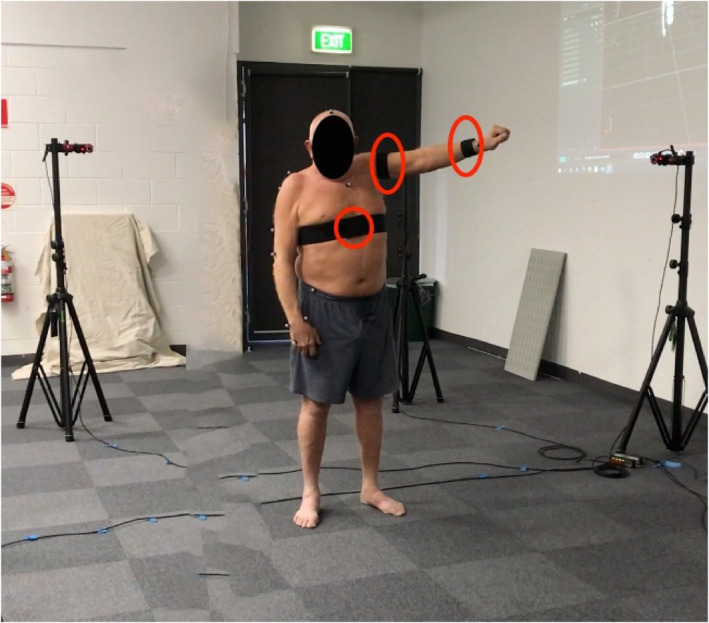
IMU Sensor setup. Circles show IMU devices fixed in place at the wrist, arm and trunk.

Participants attended the university campus where a clinical assessment of their shoulder pain and anthropometry measures were completed. The testing protocol was explained, after which participants were fitted with three IMU activity tracking sensors (Axivity Ax6). The IMU sensors were attached using an elastic strap to secure the sensor in place on the trunk (anterior lower sternum), upper arm and at the wrist on the side of their painful shoulder or their matched arm (for dominance) in the pain free group (Figure 2). The IMU sensors consist of a 3‐axis accelerometer and 3‐axis gyroscope with inbuilt memory. The accelerometer was set to record at 100 Hz with a 16g range and the gyroscope was set to 2000° per second. Sensor data were downloaded via OMGui software (Open Movement, Newcastle University, UK) at the completion of the session. The session was recorded via video camera in order to correctly label the sensor signal for accurate data analysis. Data were time synced using a unique movement and repetition pattern, before matching data points.

### 2.4 1RM Strength Measures

Participants completed a short standardised warm up (one slow set of each exercise unweighted and one set at maximum speed unweighted), followed by a one‐repetition maximum (1RM) strength test for a single arm overhead dumbbell press (shoulder press), single arm dumbbell lateral shoulder raise (lateral raise) and single arm bent over row (bent over row). Participants were provided with 1–3 min rest between each effort. Three to five progressively loaded submaximal efforts of 1–3 repetitions were completed to determine the 1RM maximum according to previously published protocols (Schroeder et al. 2007; Levinger et al. 2009; Faigenbaum et al. 2012; Baechle et al. 2008). Participants were then instructed in varying order to complete each exercise (shoulder press, lateral raise and bent over row) at 40%, 60% and 80% of their 1RM maximum result. Participants were instructed to complete each repetition as fast as they could until they were no longer able to lift with adequate form or they reported pain that prevented them from completing the exercise. Each participant then completed a standardised cool down of each exercise without additional weight. Minimal cues were provided regarding exercise form in order to mimic the most realistic conditions outside of the university laboratory.

### 2.5 Data Analysis

**FIGURE 3:**
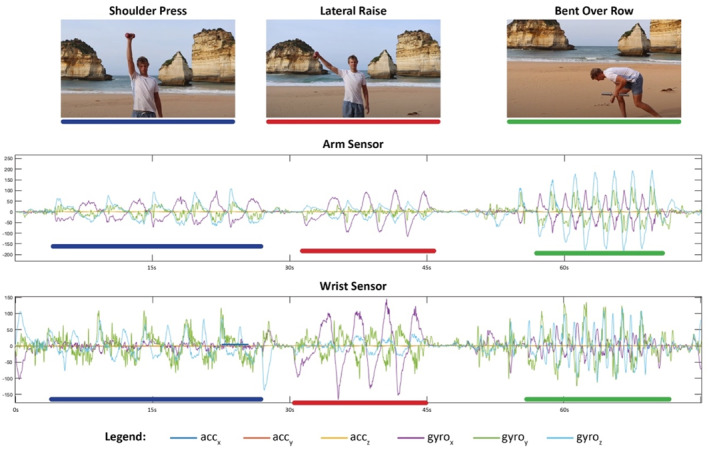
Example of raw IMU recorded signal for each exercise.

Ground truth exercise labels (3 classes, shoulder press, lateral raise or bent over row) were applied to the data manually by reviewing the video recording of each session. Raw inertial sensor data were downloaded via OMGui (Open Movement, Newcastle University, UK) and subsequently imported into a python environment for further analysis. Figure 3, shows an example of raw IMU sensor signal plotted to demonstrate each exercise.

Exercise classification was implemented in Python via the Scikit‐learn machine learning package (Pedregosa et al. 2011). Data were first filtered via a lowpass butterworth filter (20 Hz cutoff, order 5) before segmenting the data series via a 2 s sliding window. We utilised a supervised machine learning model comparing four common machine learning algorithms. These algorithms were chosen due to their use in a similar trial (Um et al. 2017; D. M. Burns et al. 2018; Bavan et al. 2019) and to test simpler algorithms before those that are more intensive or advanced. Three algorithms were the same for both a subject‐independent and subject‐dependent analysis; support vector machine with rbf kernel (SVM), random forest (RF) with *n*_estimators 500 and max depth 11 and multi‐layer perception classifier (MLP) (layer size 32.64). In addition, we used a convolutional neural network (CNN) (layer size 128, activation function dense: softmax) algorithm for subject‐independent analysis and a k‐nearest neighbours classifier (KNN) (2 nearest neighbours) for subject‐dependent analysis. SVM, RF, MLP and KNN all used a labelled encoding approach, whereas CNN used a one‐hot encoding approach (Potdar et al. 2017). 70 percent of data were used to train the model which was then tested against the remaining 30% (e.g., subject‐dependent model used 70% of data per participant for training and testing on the remaining 30% of data). For the subject‐dependent model, results were calculated based on each individual subject's data, then the mean scores of all subjects (and SD) were reported. Whereas for the subject‐independent model, accuracy was trained on the whole data set (70%) and tested against the next segment.

Of the 30% of data used to test the model, 15% of data were tested via k‐cross fold validation (valid accuracy) and 15% of the data were tested as a percentage calculation for accuracy (test accuracy). Lastly, we calculated F1 scores, a measure of the harmonic mean of the model's precision and recall. Precision is a measure of the false positives and recall is a measure of the false negatives (Grandini et al. 2020). The model was tested comparing the accuracy of a combined 3‐sensor system (trunk, arm and wrist), with the individual accuracy of the wrist, arm or trunk sensor. Lastly, we compared accuracy between participants with rotator cuff tendinopathy and matched participants without shoulder pain (separate models were trained for each group).

### 2.6 Statistical Analysis Descriptive Data

Descriptive statistical analysis was planned for all continuous outcomes. Normality was assessed for each measure using a Shapiro‐Wilk test to determine the appropriate significance test (parametric or non‐parametric) (Mishra et al. 2019). An independent samples two‐sided Welch *t*‐test was used to compare baseline characteristics and 1RM strength test results between the rotator cuff tendinopathy group and the matched controls for parametric data (West 2021). A Mann‐Whitney *U*‐test was used for non‐parametric data (West 2021). Significance was considered *p < 0.05*. All tests were conducted using R (R: A languag and e and environment for statistical computing 2020).

## 3 Results

Participant characteristics and demographics are summarised below in Table 2. Waist circumference measure was the only baseline outcome with a statistically significant difference between the groups (*p* value = 0.04, two sample *t*‐test). For the 1RM strength test results, the lateral shoulder raise showed a statistically significant difference between groups (see Table 3).

**TABLE 2:** Mean sociodemographic and anthropometric characteristics of participants.

Baseline characteristic	Rotator cuff tendinopathy mean (SD) (*n =* 18)	Pain free shoulder control mean (SD) (*n =* 18)	All mean (SD) (*n =* 36)	*p* value
Age^a^ (years)	54.2 (13.2)	54.3 (12.9)	54.3 (13.1)	0.96
Sex				
Female	9	9	18	—
Male	9	9	18	—
Anthropology				
Height (cm)	170.2 (9.6)	173.8 (8.5)	172.0 (9.2)	0.25
Weight (kg)	88.8 (17.5)	81.4 (9.0)	85.1 (14.4)	0.13
Waist (cm)*	98.6* (15.6)	88.9* (10.2)	93.8 (14.1)	0.04*
BMI^a^	30.8 (6.3)	27.1 (4.0)	28.9 (5.6)	0.05
Hand dominance				
Right	15	18	33	—
Left	3	0	3	—
Painful shoulder				
Right	14	—	—	—
Left	4	—	—	—
Pain duration (years)	5.4 (6.0)	—	—	—
Pain (NRS past week)	4.0 (2.4)	—	—	—
SPADI				
Pain total	45.9 (21.6)	—	—	—
Disability total	28.2 (21.3)	—	—	—
Total SPADI	35.0 (20.2)	—	—	—

Abbreviations: 1RM, One repetition maximum; SD, standard deviation.

^*^ *p* < 0.05.

^a^ non‐parametric test.

**TABLE 3:** Mean 1RM shoulder strength tests.

1RM strength tests (kg)	Rotator cuff tendinopathy mean (SD) (*n =* 18)	Pain free shoulder control mean (SD) (*n =* 18)	All mean (SD) (*n =* 36)	*p* value
Shoulder press	12.8 (7.2)	14.5 (5.0)	13.7 (6.2)	0.19
Lateral raise*	6.4 (3.4)	8.4 (2.3)	7.4 (3.1)	0.01*
Bent row	27.9 (12.8)	30.7 (8.4)	29.3 (10.8)	0.18

Abbreviations: 1RM, One repetition maximum; SD, standard deviation.

^*^ *p* < 0.05.

### 3.1 Accuracy of Different Machine Learning Models

Results of the machine learning models are summarised in Table 4 (subject‐independent analysis) and Table 5 (subject‐dependent analysis). Of the machine learning algorithms tested, a convolutional neural network (CNN) produced the best results for the subject‐independent data, while the random‐forest (RF) algorithm was best for the subject‐dependent data.

**TABLE 4:** Machine learning subject‐independent classification results.

	Rotator cuff tendinopathy			Pain free			All		
	Valid accuracy % (SD)	Test accuracy % (SD)	F1 (SD)	Valid accuracy % (SD)	Test accuracy % (SD)	F1 (SD)	Valid accuracy % (SD)	Test accuracy % (SD)	F1 (SD)
Wrist									
SVM	75.52 (18.4)	79.51 (19.2)	0.80 (0.18)	79.28 (16.0)	83.23 (12.8)	0.81 (0.142)	78.16 (17.6)	84.08 (16.3)	0.85 (0.125)
RF	75.4 (23.9)	80.36 (19.6)	0.81 (0.185)	79.79 (24.3)	80.87 (14.1)	0.79 (0.168)	78.46 (44.0)	85.61 (17.6)	0.87 (0.14)
MLP	76.85 (33.3)	67.86 (11.4)	0.77 (0.188)	76.91 (21.1)	70.57 (18.0)	0.70 (0.184)	77.11 (16.6)	79.85 (14.4)	0.81 (0.111)
CNN	88.2 (6.5)	88.55 (4.5)	0.85 (0.08)	90 (7.5)	90.1 (5.5)	0.89 (0.07)	92.2 (6.5)	95.55 (4.5)	0.89 (0.089)
Arm									
SVM	74.64 (20.2)	76.87 (23.5)	0.76 (0.236)	76.49 (17.2)	77.53 (14.8)	0.75 (0.165)	77.29 (17.3)	82.96 (16.9)	0.83 (0.147)
RF	72.52 (27.9)	80.36 (19.6)	0.81 (0.185)	71.78 (21.4)	69.97 (24.9)	0.72 (0.223)	79.78 (23.9)	79.56 (16.9)	0.81 (0.149)
MLP	76.85 (33.3)	75.92 (19.6)	0.77 (0.188)	66.92 (31.2)	65.60 (21.6)	0.66 (0.206)	75.11 (26.7)	77.93 (15.2)	0.79 (0.129)
CNN	89 (3.5)	90.35 (4.5)	0.9 (0.04)	90.3 (4.5)	91.5 (4.3)	0.90 (0.05)	93.2 (5.5)	93.51 (4.5)	0.90 (0.08)
Trunk									
SVM	68.85 (15.3)	67.65 (11.4)	0.66 (0.117)	74.08 (13.9)	58.66 (20.5)	0.58 (0.192)	68.23 (19.1)	63.00 (17.0)	0.63 (0.158)
RF	76.61 (31.5)	76.05 (9.79)	0.75 (0.103)	69.62 (21.3)	60.84 (20.2)	0.59 (0.194)	61.75 (21.9)	66.42 (19.1)	0.68 (0.167)
MLP	71.30 (31.7)	60.47 (18.3)	0.66 (0.121)	64.13 (31.4)	60.47 (18.3)	0.59 (0.177)	61.06 (18.4)	64.69 (17.0)	0.64 (0.167)
CNN	78.17 (7.97)	80.57 (6.7)	0.68 (0.105)	85.2 (5.5)	87.55 (6.5)	0.86 (0.05)	86 (14.6)	87 (15.7)	0.84 (0.12)
3‐Sensor									
SVM	74.25 (18.1)	81.58 (12.9)	0.81 (0.152)	74.03 (22.8)	78.18 (24.4)	0.79 (0.223)	80.29 (12.3)	85.96 (10.9)	0.84 (0.16)
RF	78.10 (38.4)	86.75 (20.3)	0.89 (0.16)	61.20 (37.6)	80.58 (27.1)	0.82 (0.238)	78.78 (20)	81.56 (17.9)	0.81 (0.15)
MLP	75.20 (22)	80.5 (15)	0.81 (0.14)	64.13 (34.4)	77.05 (23.2)	0.78 (0.211)	77.11 (22.2)	80.93 (15.2)	0.80 (0.14)
CNN	90.20 (5.4)	93.05 (4.5)	0.90 (0.056)	91.20 (4.6)	94.55 (5.5)	0.90 (0.06)	93.00 (5.1)	94.00 (4.5)	0.93 (0.045)

Abbreviations: 3‐sensor = combined wrist, arm and trunk sensor; CNN, convolutional neural network layer size [128], activation_function_dense, softmax; MLP, Multi‐layer perceptron classifier, layer size [32.64]; RF, Random forrest (n_estimators_500_max_depth_11); SVM, support vector machine with rbf kernel.

**TABLE 5:** Machine learning subject‐dependent classification mean results.

	Rotator cuff tendinopathy			Pain free			All		
	Valid accuracy % (SD)	Test accuracy % (SD)	F1 (SD)	Valid accuracy % (SD)	Test accuracy % (SD)	F1 (SD)	Valid accuracy % (SD)	Test accuracy % (SD)	F1 (SD)
Wrist									
SVM	88.57 (8.19)	91.63 (3.39)	0.90 (0.06)	87.98 (13.50)	90.01 (14.26)	0.88 (0.15)	88.28 (11.01)	90.92 (10.25)	0.89 (0.11)
RF	91.91 (4.25)	93.47 (3.04)	0.91 (0.08)	86.51 (14.06)	90.33 (12.92)	0.88 (0.15)	89.21 (10.60)	91.90 (9.39)	0.90 (0.12)
MLP	87.70 (5.31)	92.13 (3.61)	0.89 (0.08)	85.67 (14.83)	88.85 (12.53)	0.86 (0.14)	86.69 (11.03)	90.49 (9.23)	0.88 (0.11)
KNN	87.47 (7.10)	89.81 (5.22)	0.87 (0.08)	85.56 (13.26)	88.21 (13.08)	0.86 (0.14)	86.51 (10.52)	89.01 (9.85)	0.87 (0.11)
Arm									
SVM	88.95 (6.21)	92.05 (3.78)	0.90 (0.06)	85.62 (13.75)	88.18 (13.51)	0.86 (0.14)	87.29 (10.66)	90.11 (9.97)	0.88 (0.11)
RF	90.25 (4.26)	93.45 (3.24)	0.91 (0.08)	85.89 (16.02)	91.32 (13.51)	0.89 (0.14)	88.07 (11.76)	92.38 (9.40)	0.90 (0.12)
MLP	89.24 (6.69)	91.23 (4.45)	0.89 (0.08)	85.12 (11.96)	89.46 (13.30)	0.87 (0.14)	87.18 (9.78)	90.35 (9.81)	0.88 (0.11)
KNN	88.09 (6.16)	90.31 (4.93)	0.87 (0.08)	85.71 (13.22)	88.07 (13.07)	0.86 (0.14)	86.90 (10.24)	89.19 (9.80)	0.86 (0.12)
Trunk									
SVM	89.17 (7.09)	92.21 (3.40)	0.90 (0.06)	86.06 (13.21)	90.06 (13.08)	0.89 (0.14)	87.61 (10.57)	91.13 (9.48)	0.90 (0.10)
RF	91.75 (3.50)	93.72 (3.22)	0.91 (0.09)	86.11 (12.58)	90.86 (12.97)	0.89 (0.15)	88.92 (9.54)	92.28 (9.43)	0.90 (0.12)
MLP	89.02 (5.13)	92.49 (3.67)	0.90 (0.07)	85.49 (14.31)	89.48 (13.72)	0.87 (0.15)	87.25 (10.74)	90.98 (10.02)	0.89 (0.12)
KNN	87.63 (7.20)	89.10 (5.11)	0.86 (0.07)	86.51 (14.91)	88.34 (13.58)	0.86 (0.15)	87.06 (11.55)	88.71 (10.12)	0.86 (0.12)
3‐Sensor									
SVM	93.73 (2.99)	94.76 (2.51)	0.93 (0.07)	89.29 (14.79)	91.67 (13.26)	0.89 (0.16)	91.51 (10.76)	93.22 (9.53)	0.91 (0.11)
RF	95.30 (2.85)	96.12 (2.89)	0.94 (0.08)	91.74 (12.12)	94.66 (11.11)	0.92 (0.16)	93.52 (8.86)	95.39 (8.03)	0.93 (0.13)
MLP	92.58 (5.62)	95.32 (2.37)	0.94 (0.07)	90.82 (12.22)	92.99 (11.64)	0.91 (0.14)	91.70 (9.42)	94.15 (8.36)	0.92 (0.11)
KNN	91.16 (4.73)	93.62 (4.49)	0.92 (0.06)	89.98 (13.07)	92.79 (10.56)	0.90 (0.14)	90.57 (9.70)	93.21 (8.03)	0.912 (0.11)

Abbreviations: 3‐sensor, combined wrist, arm and trunk sensor; KNN, K nearest neighbour classifier_nn_2; MLP, Multi‐layer perceptron classifier, layer size [32.64]; RF, Random forrest (n_estimators_500_max_depth_11); SVM, support vector machine with rbf kernel.

### 3.2 Accuracy of Different Sensor Positions

The combined 3‐sensor system (trunk, wrist and arm) outperformed the individual sensors for the subject‐dependent results with accuracy of 95.39% (SD 8.3). Accuracy scores for the wrist, arm and trunk were 91.90% (SD 9.3), 92.38% (SD 9.40) and 92.28% (SD 9.43) respectively. For the subject‐independent data, the wrist sensor performed best with 95.55% (SD 4.5) accuracy. Accuracy scores for the trunk, arm and 3‐sensor system were 87% (SD 15.7), 93.51% (SD 4.5) and 94% (SD 4.5) respectively. Statistically significant differences were found for the subject‐independent data comparing the wrist position and the trunk position (*p = 0.003*). All other comparisons were not statistically significant.

### 3.3 Comparisons Between Rotator Cuff Tendinopathy Group and Pain Free Group

Comparing accuracy results between the rotator cuff tendinopathy group and the matched control pain free group shows results for the subject‐independent analysis were less accurate in the rotator cuff tendinopathy group for all sensor positions (93.05%, SD 4.5 compared with 94.55%, SD 5.5 for the 3‐sensor system). This may be due to greater variability within the rotator cuff tendinopathy group. Whereas, for the subject‐dependent data the reverse was true and results were more accurate in the rotator cuff tendinopathy group (96.12%, SD 2.89 compared with 94.66%, SD 11.11 for the 3‐sensor system). Neither comparison was statistically significant.

### 3.4 Accuracy for Each Exercise

**FIGURE 4:**
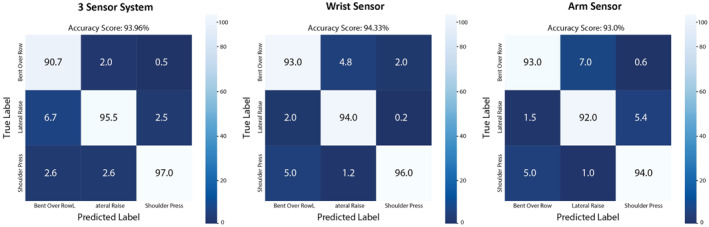
K‐Fold cross validation confusion matrix—subject‐independent model, convolutional neural network (CNN).

For the subject‐independent data, k‐fold cross validation confusion matrix results can be found in Figure 4 for the CNN algorithm. For both groups combined, best accuracy was achieved by the wrist sensor (94.33%), identifying the shoulder press with 96% accuracy, the lateral raise with 94% accuracy and the bent over row with 93% accuracy.

## 4 Discussion

This study demonstrates the accuracy of artificial intelligence, utilising machine learning to automatically classify shoulder exercise for both patients with rotator cuff tendinopathy and a matched pain free cohort. Classification accuracy is similar between a subject‐dependent model and subject‐independent model. Overall, accuracy was greater for the entire cohort with a subject‐independent model which may reflect the larger data pool available. Interestingly, utilising a single wrist based sensor achieves similar results to the 3‐sensor system (within 3%–5% accuracy) for the entire cohort using a subject‐independent analysis.

The ability to automatically detect and recognise exercise via wrist worn wearable technology presents a promising opportunity to objectively provide clinically meaningful data to clinicians and researchers, to monitor patient adherence and to provide direct patient feedback. External feedback has been demonstrated to increase patient adherence to exercise, physical activity and improve exercise technique (Ferguson et al. 2022; Scheid and West 2019; Benjaminse et al. 2015; Benjaminse and Otten 2011; Wulf 2013; Brennan et al. 2019). However, there remains a number of barriers to implement this technology from the lab into meaningful and useful clinical practice or larger research trials investigating the effects of exercise. These barriers include implementing automatic exercise detection among everyday activity, time requirements to ensure the system operates as intended and usability of the hardware or software. Integration of objective exercise monitoring in larger research studies may help better understand the various mechanisms and pathways of exercise and how they may impact patient outcomes.

Our findings are consistent with other literature findings that have investigated exercise classification and activity recognition. Several prior studies have investigated predominantly lower limb or daily activity classification via IMU sensor signal (Cheng et al. 2017; Morris et al. 2014; O'Reilly et al. 2017; Seeger et al. 2011; Clarke et al. 2017; Cleland et al. 2013; Doherty et al. 2017; Khan et al. 2013; Narayanan et al. 2020; O’Reilly et al. 2018; Parkka et al. 2007; Song et al. 2010; Stewart et al. 2018; Tavakkoli et al. 2022; Yang and Hsu 2010; Brickwood et al. 2019). There are fewer studies that have investigated upper limb or shoulder exercise classification (Um et al. 2017; D. M. Burns et al. 2018; Chang et al. 2007; Muehlbauer et al. 2011; Carnevale et al. 2019; Pan et al. 2013; Shoaib et al. 2016; Steels et al. 2020). Similarly, to our knowledge, there are only three studies that investigated exercise classification among a cohort of patients with rotator cuff tendinopathy (D. Burns et al. 2021; Bavan et al. 2019; Bavan et al. 2020).

(D. M. Burns et al. 2018) reported accuracies up to 99.4% utilising a subject‐dependent convolutional recurrent network approach and an accuracy of up to 88.9% utilising a subject‐independent approach (data outside their training set). Likewise, our own model demonstrated significantly improved results using a deep learning convolutional neural network analysis as opposed to simpler similar machine learning methods (e.g., RF, SVM or MLP algorithms). In a population of patients with rotator cuff tendinopathy, (D. Burns et al. 2021) reported exercise classification (see Supporting Information S1: Appendix for list of exercises) to be 90% (F1 score 0.82). (Bavan et al. 2019) in their lab‐based study of patients with rotator cuff tendinopathy (subacromial pain) reported 97.2% accuracy using a 10‐fold cross validation support vector machine approach. These results are comparable to our own.

A key strength of this study is the inclusion and comparison of people with rotator cuff tendinopathy, with pain free matched controls, as well as the comparison of multiple machine learning classification approaches. The main limitation of this study is the small sample of subjects and the lower average SPADI and pain scores for the participants indicating more mild limitation in pain and function. It is not clear how accurate this model will perform in a real world setting or how easily it would be to detect shoulder exercise among everyday activity. However, the minimal feedback or exercise cues provided in this study aim to replicate movement patterns closer to those likely performed in unsupervised situations.

## 5 Practical Applications

This study demonstrates the feasibility of automatic exercise classification and recognition for people completing shoulder exercises, either with rotator cuff tendinopathy or matched pain free controls. IMUs are readily accessible, affordable and available in most smartphone devices. There is potential for software development to compliment research studies to implement widespread use of this technology in monitoring exercise adherence while providing feedback regarding performance. Automated systems reduce the barriers or number of steps required to utilise such monitoring devices currently available.

This system has the potential to provide clinically meaningful data to treating practitioners either directly in person or via remote monitoring. The use of technology to provide automated exercise monitoring presents new opportunities for better understanding exercise parameters and their effect on clinical outcomes. This may lead to new metrics to monitor exercise, such as in clinical measures for rate of force development or newly developed metrics. Further, the use of technology provides an efficient means to increase scale in either research based projects or with large numbers of patients such as training athletes or monitoring gym attendees, including determining exercise variables such as volume, intensity, frequency and duration.

## 6 Conclusion

This study indicates that classifying shoulder exercise using a practical wrist worn sensor is highly accurate. Further, a similar level of accuracy was identified for subject‐independent and subject‐dependent analyses and among people with rotator cuff tendinopathy and a pain free cohort. This technology can now be used to investigate exercise recognition and classification during everyday activity and explore the effects of feedback from that information on adherence and patient outcomes.

## Funding

The authors have nothing to report.

## Conflicts of Interest

The authors declare no conflicts of interest.

## Supporting information


Supporting Information S1
