# Supplementary material for: Making Sense of Shoulder Exercise: Measuring the Accuracy of an Artificial Intelligence Model to Classify Shoulder Exercise via Wearable Sensors Among People With and Without Rotator Cuff Tendinopathy
Source: Eur J Sport Sci. 2026 Apr 8;26(5):e70167. doi: 10.1002/ejsc.70167 (PMC13060652; doi:10.1002/ejsc.70167)
Supplement: Supplementary file 1 — Supporting Information S1 [file EJSC-26-e70167-s001.docx]

## Appendix – Supplementary Data

**Supplementary Figure A1**

*Recruitment flow diagram*


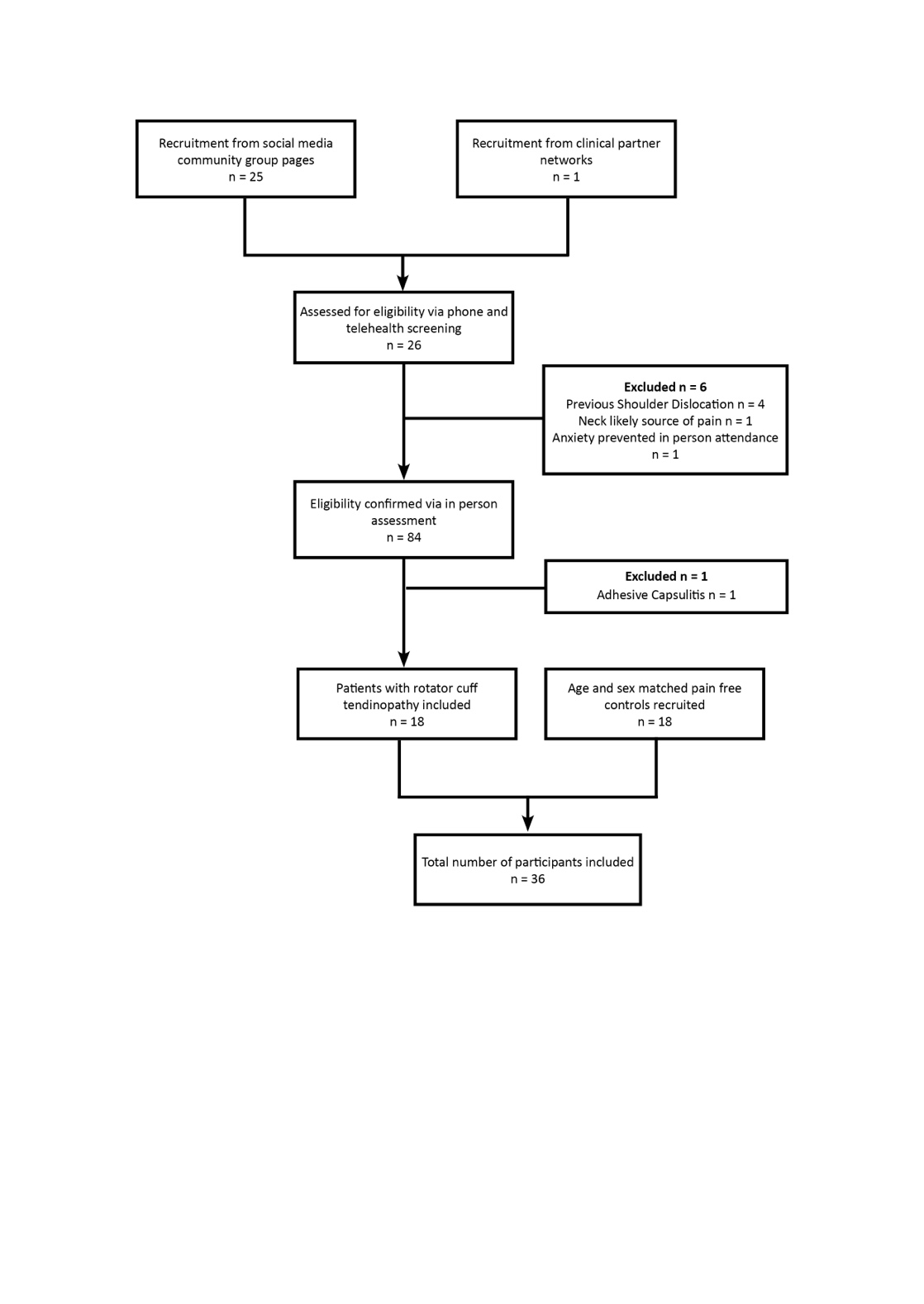


| **Supplementary Table A1**  *Inclusion and exclusion criteria for the study.* | |
| --- | --- |
| Inclusion criteria for participants with rotator cuff tendinopathy | Exclusion criteria (both groups) |
| - Primary presenting complaint of anterolateral shoulder pain (with or without referral into the upper arm) - Shoulder pain that is primarily associated with movement, particularly overhead activity or increased shoulder load resistance - Preservation of passive shoulder range of movement - Age 18 years or older | - Cervical spine referred pain - Hemiplegic shoulder pain - Systemic pathology including inflammatory joint conditions and neoplastic disorders e.g. rheumatoid arthritis - Neurological disease affecting the shoulder (e.g. Parkinson’s Disease) - Clinical diagnosis consistent with adhesive capsulitis or glenohumeral osteoarthritis |

**Supplementary Figure A2**

*IMU Sensor setup*

*Circles show IMU devices fixed in place at the wrist, arm and trunk.*


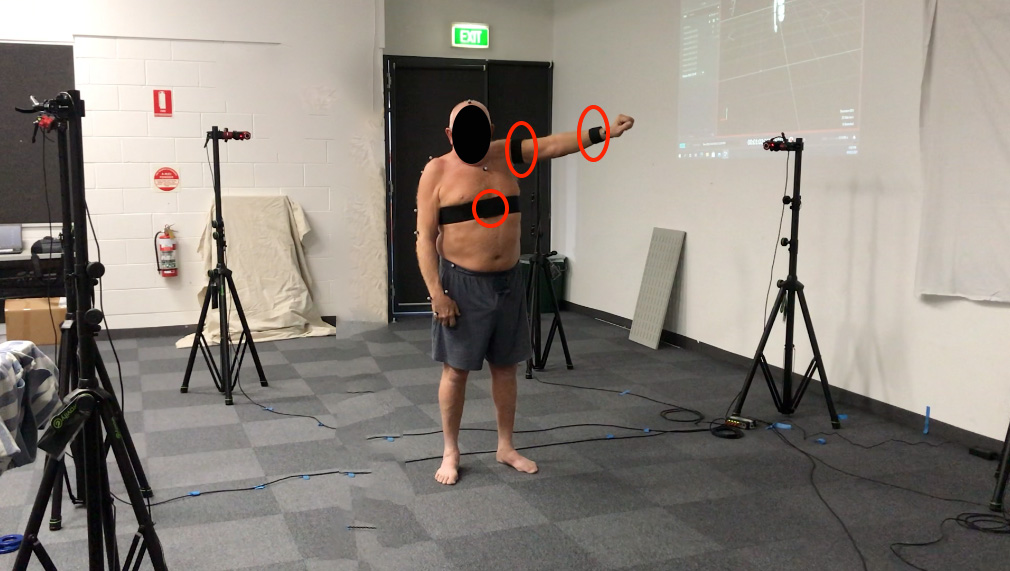


**Supplementary Table A2**
*Mean 1RM shoulder strength tests.*

| **1RM Strength Tests (kg)** | **Rotator Cuff Tendinopathy**  Mean (SD)  (*n =* 18) | **Pain Free Shoulder Control**  Mean (SD)  (*n =* 18) | **All**  Mean (SD)  (*n =* 36) | **p Value** |
| --- | --- | --- | --- | --- |
| Shoulder Press | 12.8  (7.2) | 14.5  (5.0) | 13.7  (6.2) | 0.19 |
| Lateral Raise* | 6.4  (3.4) | 8.4  (2.3) | 7.4  (3.1) | 0.01* |
| Bent Row | 27.9  (12.8) | 30.7  (8.4) | 29.3  (10.8) | 0.18 |

*Note.* SD = standard deviation; 1RM = One repetition maximum; **p*<.05

**Supplementary Table A3**
*Machine learning subject-dependent classification mean results.*

|  | **Rotator Cuff Tendinopathy** | | | **Pain Free** | | | **All** | | |
| --- | --- | --- | --- | --- | --- | --- | --- | --- | --- |
|  | **Valid Accuracy**  **% (SD)** | **Test Accuracy**  **% (SD)** | **F1**  **(SD)** | **Valid Accuracy**  **% (SD)** | **Test Accuracy**  **% (SD)** | **F1**  **(SD)** | **Valid Accuracy**  **% (SD)** | **Test Accuracy**  **% (SD)** | **F1**  **(SD)** |
| **Wrist** |  |  |  |  |  |  |  |  |  |
| SVM | 88.57  (8.19) | 91.63  (3.39) | 0.90  (0.06) | 87.98  (13.50) | 90.01  (14.26) | 0.88  (0.15) | 88.28  (11.01) | 90.92  (10.25) | 0.89  (0.11) |
| RF | 91.91  (4.25) | 93.47  (3.04) | 0.91  (0.08) | 86.51  (14.06) | 90.33  (12.92) | 0.88  (0.15) | 89.21  (10.60) | 91.90  (9.39) | 0.90  (0.12) |
| MLP | 87.70  (5.31) | 92.13  (3.61) | 0.89  (0.08) | 85.67  (14.83) | 88.85  (12.53) | 0.86  (0.14) | 86.69  (11.03) | 90.49  (9.23) | 0.88  (0.11) |
| KNN | 87.47  (7.10) | 89.81  (5.22) | 0.87  (0.08) | 85.56  (13.26) | 88.21  (13.08) | 0.86  (0.14) | 86.51  (10.52) | 89.01  (9.85) | 0.87  (0.11) |
| **Arm** |  |  |  |  |  |  |  |  |  |
| SVM | 88.95  (6.21) | 92.05  (3.78) | 0.90  (0.06) | 85.62  (13.75) | 88.18  (13.51) | 0.86  (0.14) | 87.29  (10.66) | 90.11  (9.97) | 0.88  (0.11) |
| RF | 90.25  (4.26) | 93.45  (3.24) | 0.91  (0.08) | 85.89  (16.02) | 91.32  (13.51) | 0.89  (0.14) | 88.07  (11.76) | 92.38  (9.40) | 0.90  (0.12) |
| MLP | 89.24  (6.69) | 91.23  (4.45) | 0.89  (0.08) | 85.12  (11.96) | 89.46  (13.30) | 0.87  (0.14) | 87.18  (9.78) | 90.35  (9.81) | 0.88  (0.11) |
| KNN | 88.09  (6.16) | 90.31  (4.93) | 0.87  (0.08) | 85.71  (13.22) | 88.07  (13.07) | 0.86  (0.14) | 86.90  (10.24) | 89.19  (9.80) | 0.86  (0.12) |
| **Trunk** |  |  |  |  |  |  |  |  |  |
| SVM | 89.17  (7.09) | 92.21  (3.40) | 0.90  (0.06) | 86.06  (13.21) | 90.06  (13.08) | 0.89  (0.14) | 87.61  (10.57) | 91.13  (9.48) | 0.90  (0.10) |
| RF | 91.75  (3.50) | 93.72  (3.22) | 0.91  (0.09) | 86.11  (12.58) | 90.86  (12.97) | 0.89  (0.15) | 88.92  (9.54) | 92.28  (9.43) | 0.90  (0.12) |
| MLP | 89.02  (5.13) | 92.49  (3.67) | 0.90  (0.07) | 85.49  (14.31) | 89.48  (13.72) | 0.87  (0.15) | 87.25  (10.74) | 90.98  (10.02) | 0.89  (0.12) |
| KNN | 87.63  (7.20) | 89.10  (5.11) | 0.86  (0.07) | 86.51  (14.91) | 88.34  (13.58) | 0.86  (0.15) | 87.06  (11.55) | 88.71  (10.12) | 0.86  (0.12) |
| **3-Sensor** |  |  |  |  |  |  |  |  |  |
| SVM | 93.73  (2.99) | 94.76  (2.51) | 0.93  (0.07) | 89.29  (14.79) | 91.67  (13.26) | 0.89  (0.16) | 91.51  (10.76) | 93.22  (9.53) | 0.91  (0.11) |
| RF | 95.30  (2.85) | 96.12  (2.89) | 0.94  (0.08) | 91.74  (12.12) | 94.66  (11.11) | 0.92  (0.16) | 93.52  (8.86) | 95.39  (8.03) | 0.93  (0.13) |
| MLP | 92.58  (5.62) | 95.32  (2.37) | 0.94  (0.07) | 90.82  (12.22) | 92.99  (11.64) | 0.91  (0.14) | 91.70  (9.42) | 94.15  (8.36) | 0.92  (0.11) |
| KNN | 91.16  (4.73) | 93.62  (4.49) | 0.92  (0.06) | 89.98  (13.07) | 92.79  (10.56) | 0.90  (0.14) | 90.57  (9.70) | 93.21  (8.03) | 0.912  (0.11) |

*Note.* SVM = support vector machine with rbf kernel; RF = Random forrest (n_estimators_500_max_depth_11); MLP = Multi-layer perceptron classifier, layer size [32.64]; KNN = K nearest neighbour classifier_nn_2; 3-sensor = combined wrist, arm and trunk sensor

### **Supplementary Table A4**

### Table of Exercises used in Burns et al. (2021)

| **Exercise** | **Categorised into motion types for machine learning classification** |
| --- | --- |
| 1. Assisted shoulder flexion (lying) | flexion |
| 2. Assisted shoulder flexion (standing) | flexion |
| 3. Assisted shoulder external rotation (sitting) | ER |
| 4. Assisted shoulder internal rotation (standing) | IR |
| 5. Active shoulder flexion (standing) | flexion |
| 6. Press up against wall (standing) | press-up |
| 7. Shoulder girdle stabilization with elevation (standing) | flexion |
| 8. Resisted lat pull down (standing) | pull-down |
| 9. Resisted row (standing) | row |
| 10. Resisted external rotation (standing, adducted) | ER |
| 11. Resisted internal rotation (sitting, adducted) | IR |
| 12. Active shoulder abduction (standing) | abduction |
| 13. Assisted shoulder internal rotation (side-lying) | IR |
| 14. Resisted lat pull down (standing, external-rotation) | pull-down |
| 15. Resisted serratus anterior (sitting) | press-up |
| 16. Resisted shoulder scaption (sitting) | flexion |
| 17. Resisted triceps pull down (standing) | elbow-extension |
| 18. Resisted external rotation (standing, abducted) | ER |
| 19. Pushup | press-up |
